# TDtest: easy detection of bacterial tolerance and persistence in clinical isolates by a modified disk-diffusion assay

**DOI:** 10.1038/srep41284

**Published:** 2017-02-01

**Authors:** Orit Gefen, Betty Chekol, Jacob Strahilevitz, Nathalie Q. Balaban

**Affiliations:** 1 Racah Institute of Physics, Edmond J. Safra Campus, The Hebrew University of Jerusalem, Jerusalem 9190401, Israel; 2 Department of Clinical Microbiology and Infectious Diseases, Hadassah-Hebrew University Medical Center, Jerusalem, 91120, Israel; 3 The Harvey M. Kruger Family Center for Nanoscience and Nanotechnology, Edmond J. Safra Campus, The Hebrew University of Jerusalem, Jerusalem 9190401, Israel

## Abstract

Antibiotic tolerance - the ability for prolonged survival under bactericidal treatments - is a potentially clinically significant phenomenon that is commonly overlooked in the clinical microbiology laboratory. Recent *in vitro* experiments show that high tolerance can evolve under intermittent antibiotic treatments in as little as eight exposures to high doses of antibiotics, suggesting that tolerance may evolve also in patients. However, tests for antibiotic susceptibilities, such as the disk-diffusion assay, evaluate only the concentration at which a bacterial strain stops growing, namely resistance level. High tolerance strains will not be detected using these tests. We present a simple modification of the standard disk-diffusion assay that allows the semi-quantitative evaluation of tolerance levels. This novel method, the “TDtest”, enabled the detection of tolerant and persistent bacteria by promoting the growth of the surviving bacteria in the inhibition zone, once the antibiotic has diffused away. Using the TDtest, we were able to detect different levels of antibiotic tolerance in clinical isolates of *E. coli*. The TDtest also identified antibiotics that effectively eliminate tolerant bacteria. The additional information on drug susceptibility provided by the TDtest should enable tailoring better treatment regimens for pathogenic bacteria.

Tolerant and persistent bacteria can survive antibiotic treatments without having acquired a resistance phenotype^1^,^2^,^3^,^4^,^5^. In contrast to resistance, which enables bacteria to grow in a concentration of a drug that would otherwise prevent growth, tolerance is only a transient ability of bacteria to survive under otherwise bactericidal treatments^6^ (Fig. 1). The term “persistence” is used when this ability is found only for a small sub-population of bacteria within a clonal and susceptible population^1^. Note that “persistence” is also used more generally to describe an infection that is not cleared effectively in the host^7^, but here we restrict the term to an heterogeneous response of a clonal bacterial population to antibiotics. For example, a major mechanism of tolerance and persistence is dormancy. Dormant bacteria are killed less efficiently by many antibiotics that target active growth processes such as cell wall assembly or DNA replication^6^. Our recent results showed that intermittent exposure to a beta-lactam antibiotic treatment *in vitro* led to the rapid evolution of transient dormancy and tolerance until it reached 100% of the population. A few daily exposures for 3 hours were sufficient to evolve this high level of tolerance to the antibiotic, and led to treatment failure^8^. Interestingly, bacteria evolved to adapt to the *duration* of the transient antibiotic exposure rather than to its precise chemical nature and evaded antibiotic killing by remaining dormant for the duration of the treatment, without becoming resistant. Since the regime of antibiotic exposure *in vitro* is similar to a typical treatment in patients, detecting tolerance in the clinic may be crucial for identifying mechanisms related to survival and for devising effective treatment regimens. Moreover, studies showed that tolerance, leading to treatment failure, can rapidly evolve for other classes of antibiotics^9^, as well as during treatment^10^, and mutations related to persistence have been identified in clinical isolates^11^. Therefore, the routine detection of tolerance in clinical isolates may shed new light on pathways leading to failure of antibiotic treatment. Current Clinical Microbiology laboratory practice of analyzing antibiotic treatment failure is set to identify isolates with a Minimum Inhibitory Concentration (MIC) higher than the breakpoint. Persistent and tolerant bacteria will be overlooked because their MIC is unchanged.

The goal of this study was to provide a simple detection assay to evaluate tolerance or persistence levels in clinical isolates. Our assay is based on the Kirby–Bauer disk diffusion antibiotic susceptibility testing^12^. In this disk diffusion assay, nutrients are consumed by the growing bacteria. Consequently, the small population of tolerant bacteria which have survived the bactericidal antibiotic within the inhibitory zone, would not, however, be detected because nutrients are depleted by the time the antibiotic level drops below the MIC.

We propose the Tolerance Disk Test (TDtest), a modification of the standard Kirby–Bauer disk diffusion assay that enables, in addition to determining resistance, to identify tolerance to an antibiotic by evaluating survival.

## Results

### The standard disk-diffusion assay does not detect tolerance

**Figure 1:**
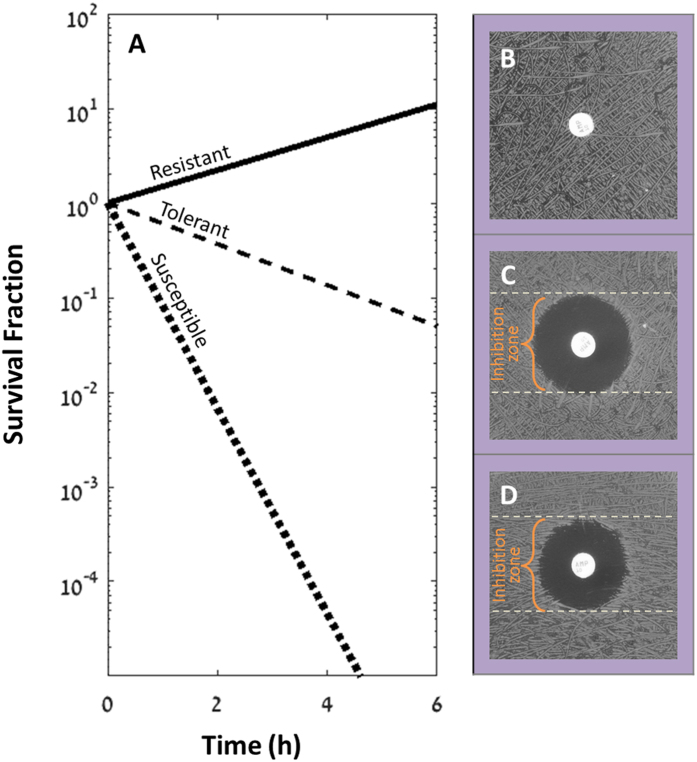
Different responses to antibiotics. (**A**) Schematic plot of the fraction of surviving bacteria in batch culture for a resistant strain (solid line), tolerant strain (dashed line) and susceptible strain (dotted line). (**B**–**D**) Examples of standard Disk diffusion assay results with ampicillin (10 μg). White dashed lines mark the diameter of the dark zone where the growth of bacteria is prevented by the antibiotics i.e. the “inhibition zone”. (**B**) Resistant strain (MGZ1-PSA11, Table 1) (MIC > 100 μg/ml). (**C**) Tolerant strain (tbl3a, MIC = 3 ± 1 μg/ml). (**D**) Susceptible strain (KLY, Table 1) with the same MIC as the strain shown in (**C**). The standard Disk diffusion assay does not distinguish between susceptible and tolerant strains. A slightly larger inhibition zone may be seen in tolerant strains because of delayed growth. Disk diameter: 6 mm.

The Kirby–Bauer disk diffusion method is designed to identify resistant bacteria by creating a gradient of concentration around a disk impregnated with antibiotics. The gradient of antibiotics prevents the growth of bacteria creating an “inhibition zone” around the disk (Fig. 1). A resistant strain (Fig. 1B) will grow closer to the disk than a susceptible strain (Fig. 1D). However, applying the disk diffusion assay to a mutant strain that evolved under 3 hours of intermittent ampicillin exposures and bearing tolerance mutation^8^ in the *vapBC* toxin-antitoxin module did not reveal any reduction of the inhibition zone (Fig. 1C) when compared to the wild-type strain (Fig. 1D).

### Understanding the diffusion process in the Kirby–Bauer disk diffusion method for tolerant bacteria

**Figure 2:**
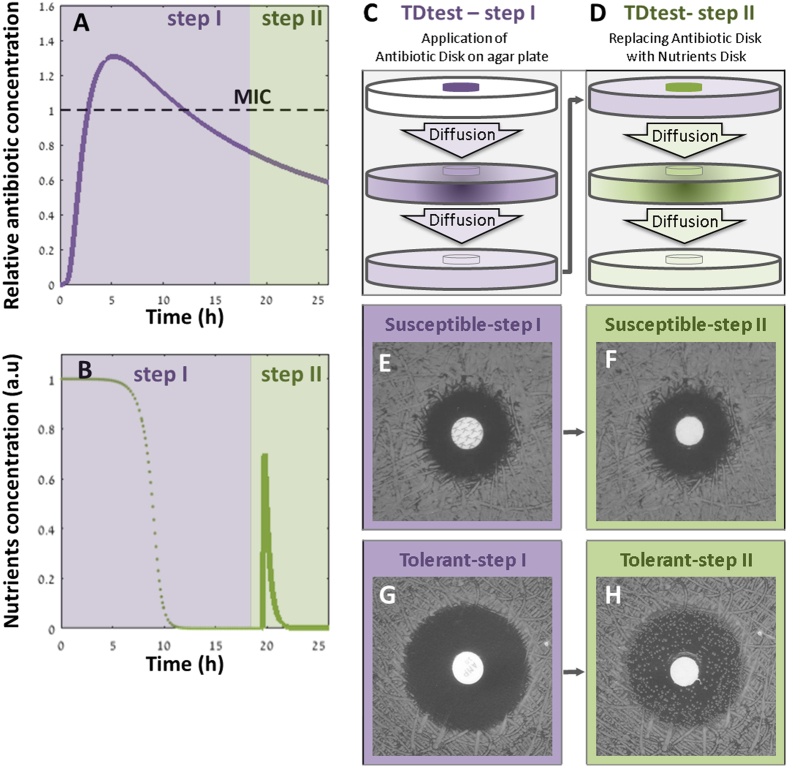
Rationale for the TDtest method for detecting differences in tolerance levels. (**A**) Theoretical antibiotic concentration profile over time at a point 0.8 cm from the center of the antibiotic disk, assuming a point-source diffusion profile (Eq. 1). As the antibiotic diffuses in the agar, the concentration increases rapidly and then slowly decreases. After several hours (here: 12 h), the antibiotic concentration falls below the MIC at this point. The concentration is shown in units of the MIC, *D* = 8.5e-6 *cm*^2^ · *sec*^−1^ (See Fig. S11) and *A* = 10 μg, *h* = 0.4 cm. See Supplementary Fig. S9 for the profile of the antibiotic in the plate over time. (**B**) Schematic concentration profile over time at the same representative point as shown in (**A**). Nutrients in the whole plate are consumed by the growing bacteria outside the inhibition zone (dotted line). Due to diffusion, nutrients in the inhibition zone are also depleted after several hours. The lack of nutrients prevents the re-growth of surviving bacteria, even though the antibiotics concentration is below the MIC. Replacing the antibiotic disk with the glucose disk in the TDtest allows re-growth and detection of the surviving bacteria (solid line). (**C**) TDtest first step: the antibiotic disk is put on top the agar plate. The antibiotic diffuses from the disk. (**D**) TDtest second step: replacing the antibiotics disk with a glucose disk. (**E**,**F**) Susceptible strain: growth inhibition around the antibiotic disk (**E**), no late growth after glucose addition (**F**). (**G**,**H**) Tolerant strain: growth inhibition around the antibiotic disk (**G**), colonies inside the inhibition zone after glucose addition, indicate slow or late growing bacteria of this strain (**H**). Disk diameter: 6 mm.

In order to understand better why the high tolerance does not appear in the standard disk-diffusion assay, one should think about both the diffusion of the antibiotic and bacterial growth on the plate. The size of the inhibition zone around the disk is set by the interplay between the diffusion rate of the antibiotic (see Equation 1 and Fig. 2), the growth rate of the bacteria and the MIC.


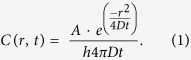


Where *C* is the concentration of antibiotics as a function of time (*t*) and distance (*r*) from the source (center of disk), assuming isotropic diffusion in a two-dimensional plate. *D* is the diffusion coefficient, *A* is the initial antibiotic amount in the disk and *h* is the height of the agar in the plate.

Depending on *A*, after overnight incubation, the antibiotic concentration in the inhibition zone can drop below the MIC (Fig. 2A). However, by this time, the nutrients have diffused in the plate and are depleted by the bacteria that grew in the inhibition zone periphery (Fig. 2B). Tolerant bacteria that have survived the transient exposure to the antibiotic would not be detected because of the lack of nutrients supporting their visible growth.

### Adding nutrients to the Kirby–Bauer disk diffusion method enables the detection of tolerant and persistent bacteria

The TDtest enables the detection of tolerant strains by overcoming the nutrient depletion that occurs in the Kirby–Bauer disk diffusion method. In Fig. 2A we schematically plot the diffusion dynamics during the TDtest in an arbitrary point in the inhibition zone. In this example, the antibiotic concentration drops to below the MIC after about 12 hours. Tolerant bacteria that managed to survive in the inhibition zone cannot grow because the nutrients were depleted (Fig. 2B, dotted line) by the bacteria growing beyond the inhibition zone. The surviving bacteria in the inhibition zone are then recovered by replacing the antibiotic disk (now emptied) with a new disk impregnated with nutrients (Fig. 2C,D). As nutrients diffuse away from the new disk, they now promote the growth of tolerant bacteria that can form detectable colonies in the inhibition zone (Fig. 2H, Supplementary Fig. S2 and Movie S1).

**Figure 3:**
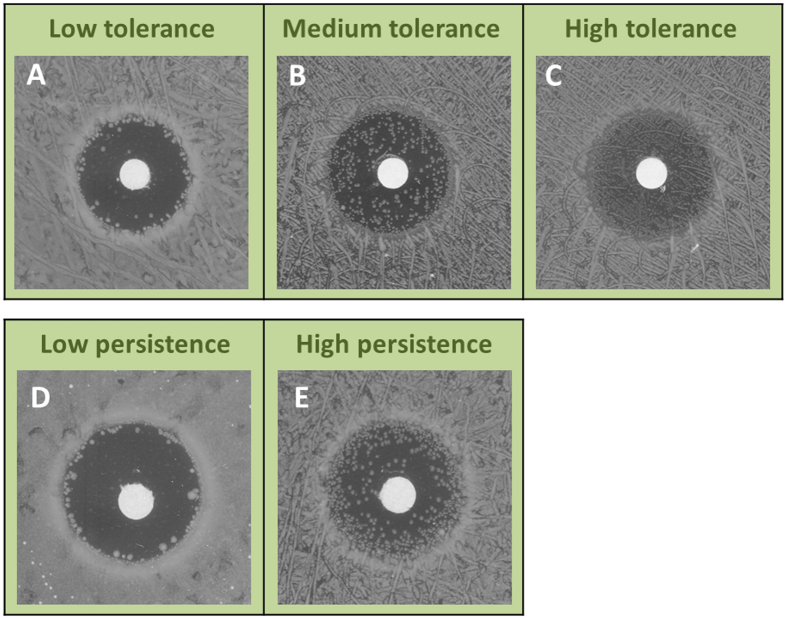
TDtest second step detects different levels of tolerance and persistence. Colonies inside the inhibition zone after the second step of the TDtest. (**A**) low-tolerance *WT* strain. (**B**) medium-tolerance *vapB* mutant strain (tbl3a). (**C**) high-tolerance *metG* mutant strain (tbl5a). (**D**) low-persistence *WT* strain (MGY). (**E**) *hipA7* high-persistence mutant (MGHY, Table 1)^13^,^14^. For more controls, See Supplementary Figs S1, S5 and S6. Disk diameter: 6 mm.

Thus, whereas the standard assay shows a similar inhibition zone for a susceptible (Fig. 1D) and a tolerant strain (Fig. 1C), the TDtest discriminates between susceptible and tolerant strains by uncovering the surviving bacteria of the latter (Fig. 2F and H). Furthermore, a qualitative evaluation of tolerance can be done by the number of colonies inside a typical inhibition zone (radius ~1 cm): “low tolerance” (0–10 colonies in inhibition zone), “medium tolerance” (10- to a few hundreds colonies in inhibition zone) and high tolerance (bacterial lawn in inhibition zone) (Fig. 3A–C) in *wt* K-12, tbl3b and tbl5a strains, respectively^8^ (Table 1).

The TDtest can detect also sub-populations of tolerant bacteria, namely persistent bacteria. For example, strains bearing the *hipA7* mutations have high persistence^13^,^14^, due to hyperactivation of the stringent response that results in the transient growth arrest of a sub-population of bacteria^15^. Whereas the majority of the *hipA7* population is killed by beta-lactams as efficiently as the *wt* strain, a sub-population of tolerant bacteria that require significantly more time to be killed can be observed. The coexistence of these two populations, normal bacteria and tolerant “persister” bacteria, results in a bimodal killing curve, the hall mark of persistence^6^. The TDtest detects high tolerance to a beta-lactam in the *hipA7* mutant (Fig. 3D,E). Note that the TDtest evaluates the tolerance level semi-quantitatively and therefore does not distinguish between tolerance and persistence.

**Figure 4:**
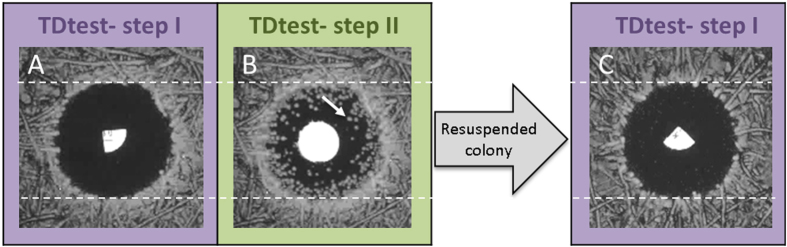
Colonies detected with the TDtest are typically not due to resistant mutants. (**A**) The inhibition zone after the first step of the TDtest (exposure to antibiotics only- Imipenem 2.5 μg). The dashed lines mark the diameter of the inhibition zone. (**B**) The inhibition zone after the second step of the TDtest (replacement of the antibiotic disk with a glucose-containing disk). Appearance of colonies inside the inhibition zone occurs after a few hours and indicates tolerant/persistent bacteria. (**C**) A colony that grew inside the inhibition zone (panel B, white arrow) was picked and retested for the same antibiotic after overnight growth. The dashed lines mark the diameter of the inhibition zone, same as in panel A. Similar results were obtained on more colonies and also for ampicillin (Supplementary Fig. S3), see also Supplementary Fig. S4. Disk diameter: 6 mm.

In all the cases mentioned above, the tolerant colonies that appear following the addition of the nutrient disk are typically not resistant. When the progeny of a colony that grew inside the inhibition zone is exposed to the disk diffusion assay with the same antibiotic, the inhibition zone radius remains unchanged, i.e. these colonies are not more resistant (Fig. 4 and Supplementary Fig. S3).

The tolerant bacteria detected with the TDtest are also distinct from heteroresistance, that may also result in colonies inside the inhibition zone. Heteroresistant colonies would appear already during the first step of the TDtest, as they are detected in the standard disk assay^16^.

### TDtest reveals different tolerance levels in clinical isolates

**Figure 5:**
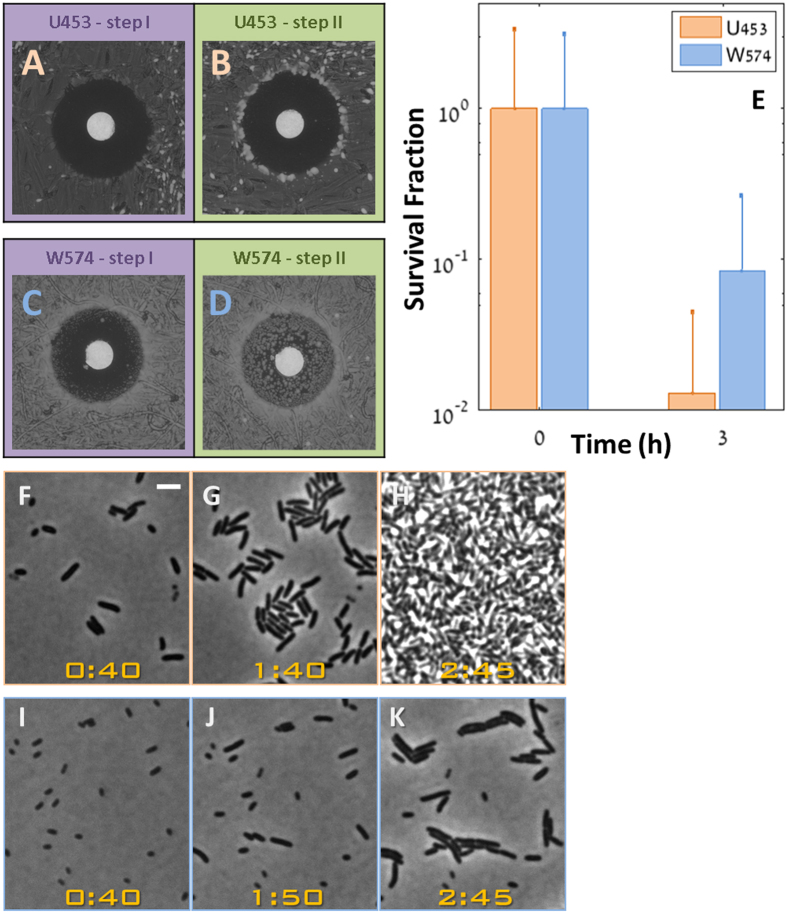
TDtest reveals different tolerance levels in clinical isolates exposed to the same antibiotic. TDtest for *E. coli* strains U453 (**A**,**B**) and W574 (**C**,**D**) with ertapenem (0.25 μg). (**A**) U453 after standard exposure to ertapenem disk. (**B**) U453 at the end of TDtest. The absence of colonies inside the inhibition zone indicate that this strain has a low tolerance level (**C**) W574 after standard exposure to ertapenem disk. (**D**) W574 at the end of TDtest. The high number of microcolonies growing inside the inhibition zone indicates that this strain has a high tolerance level. (**E**) Survival fraction of W574 (Blue) and U453 (orange) under ertapenem (10 μg/ml) showing the slower killing of the W574 tolerant strains. (**F**–**K**) Time-lapse microscopy of the strains shown in (**A**–**E**) without antibiotics, reveals that the tolerance is due to an extended lag phase of the high tolerance strain: (**F**–**H**) U453. (**I**–**K**) W574. At *t* = 40 min, the short lag strain (W453) has already started to grow. Therefore the bacteria are bigger. (Bar: 3 μm, Time: hours:min).

Even carbapenems, one of the broadest-spectrum class of antibiotics, are not free of tolerance. Therefore, for clinical isolates resistant to ampicillin, we applied the TDtest with ertapenem. We were able to discriminate by tolerance level two ertapenem susceptible clinical isolates of *E. coli* (MIC < 0.05 μg/ml) (Table 1). Strain U453 (Fig. 5A,B) shows low tolerance, whereas strain W574 (Fig. 5C,D) shows high tolerance. Measurement of the fraction of survival of a liquid culture under ertapenem at a concentration of 10 μg/ml (similar to the mean serum concentration at 12 hours after 1 gram dosing)^17^, corroborate the TDtest results, namely the higher tolerance under ertapenem of W574 versus U453 (Fig. 5E). Moreover, direct monitoring by time-lapse microscopy shows that the difference in tolerance levels measured with the TDtest correlates with the cell-to-cell variability in the initiation of growth of the two strains in the absence of antibiotics. Whereas the low tolerance strain, U453, grows very uniformly and rapidly (Fig. 5F–H), cells of the medium tolerance strain, W574, vary in the time of initiation of growth (Fig. 5I–K). Because beta-lactams target effectively only growing bacteria, the bacteria that initiated growth late would have a higher probability of surviving the ertapenem treatment^8^, namely their tolerance level is higher. Medium tolerance was also detected in a clinical isolate of *Enterobacter cloacae* (Supplementary Fig. S2).

The TDtest was evaluated also for bacteria grown on other media typically used in the clinical microbiology, namely Mueller-Hinton agar and Mueller-Hinton blood agar (Supplementary Fig. S8).

### Easy detection of antibiotics effective against tolerant bacteria with the TDtest

**Figure 6:**
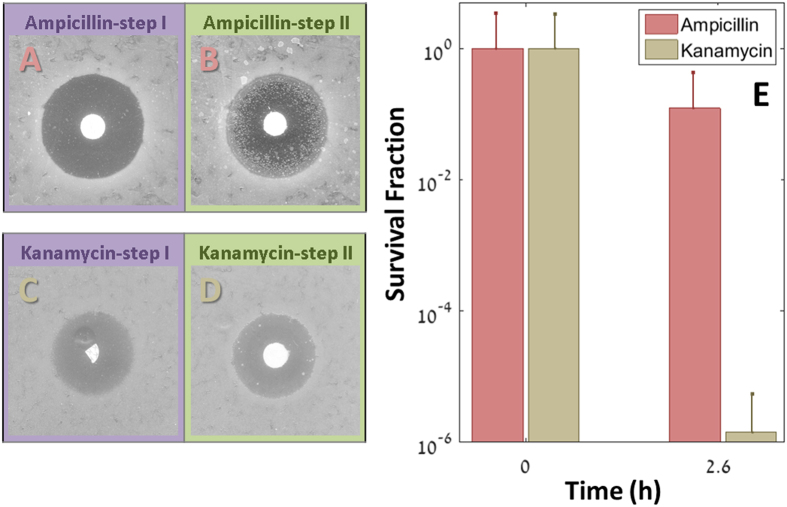
The TDtest identifies antibiotics that eliminate tolerant bacteria. (**A**,**B**) Observation of tolerance to ampicillin (10 μg) in tbl3a. (**C**,**D**) Same strain (tbl3a) exposed to Kanamycin (7.5 μg). No tolerance is observed (See Fig. S10 for empirical determination that the kanamycin concentration drops below the MIC) Disk diameter: 6 mm. (**E**) Time-kill curves of the same strain in Kanamycin 100 μg/ml (beige) and Ampicillin 100 g/ml (red). The large fraction of survivals in Ampicillin (12%, after 2.6 hours) was predicted by the late colonies appearance, and is absent in Kanamycin (1.4E-4%, after 2.6 hours).

Using the TDtest, we were able to screen for antibiotics more effective against tolerant bacteria. We compared the effect of different antibiotics on the same *E. coli* tolerant strain tbl3a, obtained by evolution under intermittent exposure to ampicillin^8^. The results are shown in Fig. 6A–D: the bacteria show medium tolerance to ampicillin (Fig. 6A,B), but low tolerance to kanamycin (Fig. 6C,D).

We verified this finding by measuring survival in liquid culture after exposure to the two antibiotics. An overnight culture was diluted and exposed either to ampicillin (100 μg/ml) or kanamycin (100 μg/ml). In accordance to the results of the TDtests, the survival fraction under ampicillin was three orders of magnitude higher than under kanamycin (Fig. 6E). Thus, although the “regular” disk-diffusion test did not point to an advantage of kanamycin over ampicillin against this strain (Fig. 6A and C), the TDtest revealed the potentially higher effectiveness of kanamycin against the tolerant bacteria (for more antibiotics, See Supplementary Fig. S6).

## Discussion

Late-growing bacteria, leading to tolerance or persistence, may survive an antibiotic treatment and might lead to treatment failure^18^. The level of tolerance of an isolate to a particular drug is, however, not taken into account when choosing antibiotics. At present, a major limitation of addressing the phenomenon of tolerance in the clinic is the absence of a simple detection technique for tolerant strains. In this work, we introduce a cheap and simple “upgrade” to the Kirby-Bauer disk diffusion test. The first part of the test, similar to the standard disk diffusion test, detects resistance. For strains that do not manifest resistance, a second step evaluates their level of tolerance by replacing the antibiotic disk with a second disk, impregnated with nutrients only. Surviving bacteria present in the inhibition zone will then be detected.

Slower killing rate of tolerant bacteria may not always be relevant to treatment outcome; preventing bacterial growth is often sufficient because the immune system will be able to clear the infection. For example: for patients with large bacterial burdens, such as nosocomial pneumonia, it is imperative to kill ≥2 log10 CFU/g early after treatment initiation, to allow the granulocytes to contribute optimally to bacterial clearance^19^. However, in infections that are not cleared efficiently by the immune system, either because of immunodeficiency or because of difficulty in accessing the site of infection, such as infective endocarditis, tolerance may have strong implications on treatment outcome^20^. Integration of the TDtest in the Clinical Microbiology laboratory could thus be a valuable tool to facilitate the study of the clinical implication of tolerance and persistence.

A limitation of the TDtest is its sensitivity to the growth conditions, as tolerance and persistence phenotypes are often triggered by external conditions, such as tolerance that requires passage through to stationary phase conditions^6^ or tolerant forms that require the host environment^21^. Such limitation could be addressed in the future by testing several growth conditions, for example by mimicking the host environment better than the standard media currently used *in vitro*.

In addition to its potential benefit for the clinical setting, the TDtest may enable screens for compounds or combinations that are potent against tolerant and persistent bacteria and speed up drug development. Finally, the simplicity and low cost of the TDtest technique may be used in third world country to characterize strains and classify them according to the rapidity of their killing under several antibiotics.

## Material and Methods

### Media and reagents

Growth medium was LB Lennox (LBL) and all other chemicals, unless stated otherwise, were purchased from Sigma Chemical Co. Ampicillin stock solution 100 mg/ml in Double Distilled Water (DDW) was kept as single use aliquots at −20 °C. Ertapenem (MERCK) stock solution (20 mg/ml in DDW) was kept at −20 °C. Kanamycin stock solution (30 mg/ml in DDW) was kept at −20 °C.

Antibiotic content in disks: ampicillin 10 μg (Bio-rad) and kanamycin 7.5 μg (Bio-rad), ertapenem 0.25 μg, cefazolin 7.5 μg (OXOID), ciprofloxacin 0.1 μg, imipenem 2.5 μg (OXOID).

Glucose (D-glucose) was purchased from JT Baker.

Mueller-Hinton (MH) plates and MH + 5% sheep blood (MH-BD) agar plates were purchased from Novamed.

### TDtest

The TDtest consists of two steps:

Step I: similar to reference disk diffusion assay^22^, with the following exceptions:

Circa 10^6^–10^7^ bacteria at stationary phase were plated on LBL nutrient agar plate.

The amount of antibiotics in the disks was adjusted to reach a concentration below the MIC in the inhibition zone after a day of incubation (at 37 °C). Many commercial disks contain amounts that are too high for the concentration to fall below the MIC within the inhibition zone after a day. When this was the case, custom disks for the TDtest were prepared (see below).

If the MIC is not known, preparing several disks that contain different amounts of antibiotics and performing the TDtest in parallel for those disks is recommended (See Supplementary Fig. S7). We found that the optimal amount of antibiotic for the TDtest should be between the amount used in the commercial disk, and above 1/50 of that amount.

Step II: The antibiotic disk was replaced with a glucose (2 mg) disk after 18 hours and the plate was incubated for an additional overnight.

Alternatives for this step:

- Adding the glucose solution directly on the antibiotic disk, instead of replacing it.
- Using other nutrients instead of glucose.

### Preparation of disks

Filter paper (Whatman, #1) were cut in circles of 6 mm diameter, sterilized by autoclave and impregnated with either 5 μl of a 40% sterile glucose solution for glucose disks, or 4–10 μl of antibiotic solution, according to final required amount for antibiotic disks, and left to dry at room temperature. Alternatively, commercial disks were cut in half or quarter to reduce the amount. Note that the exact amount of antibiotics is not important as long as it is high enough to create a large inhibition zone, and low enough to allow the concentration to fall below the MIC after 18 hours. The amount of antibiotic in each disk shown in the figures, is mentioned in the legend.

### Time-lapse imaging of plates

Imaging of plates was done using an automated multiple scanner system (ScanLag), as described in ref. 23.

### Measurement of diffusion coefficient

To measure the diffusion coefficient we used a one-well rectangular plate (12*7.5 cm), filled with agar 1.5%, to a height of 0.4 cm (similar to the plates used for the TDtest assays). We added a drop of a fluorescent agent (fluorescein, with molecular weight of 332 g/mol, which is close to ampicillin 349 g/mol) at a single point in the middle of the plate, and measured the temporal and spatial dynamics of fluorescence over time at 37 °C, for 672 points in the plate, using multiplate reader Infinite M200 (Tecan). The diffusion coefficient estimate was extracted by fitting the data to the 2D diffusion equation (Eq. 1) (See Supplemental Fig. S11).

### Time-kill experiments

Overnight cultures were diluted 1:100 in fresh medium containing antibiotic and incubated at 37 °C with shaking at 270 rpm, for designated time. Bacterial survival was determined by the most probable number-counting method (MPN)^24^.

### Time-lapse microscopy

Time-lapse microscopy was performed as in ref. 13. Briefly, a Polydimethylsiloxane (PDMS) square mold was cut out of cured Sylgard184 (Dow Corning) layer (thickness: about 3 mm). The mold was filled with melted LBL agarose 1.5%. Bacteria (about 5 μl of 1:10 from stationary phase) were put on a coverslip (#1.5) and covered with the solidified LB-agarose inside the PDMS mold. The whole chamber was sealed with another coverslip to avoid agarose drying. The PDMS chambers were monitored using a Leica DMIRBE inverted microscope system with incubator box (Life Imaging Systems) at 37 °C, automated stage and shutters. Autofocus and image acquisition was done using Micro-Manager^25^ to control the microscope, stage, shutters and camera. Multiple locations were monitored in parallel for phase-contrast imaging. Images were acquired using a 100× oil objective and a CCD camera (Orca-ER; Hamamatsu).

### Antibiotics susceptibility tests

Each column in a 96 wells plate was filled with fresh LBL supplemented with ascending amount of ampicillin and inoculated with approx. 10^4^ bacteria/well. The plate was incubated overnight at 37 °C. The Minimal Inhibitory Concentration (MIC) is the highest concentration supporting visible growth.

## Additional Information

**How to cite this article:** Gefen, O. *et al*. TDtest: easy detection of bacterial tolerance and persistence in clinical isolates by a modified disk-diffusion assay. *Sci. Rep.* **7**, 41284; doi: 10.1038/srep41284 (2017).

**Publisher's note:** Springer Nature remains neutral with regard to jurisdictional claims in published maps and institutional affiliations.

## Supplementary Material

Supplementary Figures and Movie

Supplementary Movie S1

## Figures and Tables

**Table 1:** Bacterial strains.

Strain	Relevant characteristics	Source
W574	*E. coli* clinical strain isolated from a wound infection	This work
U453	*E. coli* clinical strain isolated from a urine infection	This work
B340	*E. cloacae* clinical strain isolated from a bloodstream infection	This work
KLY	*E. coli* K-12 strain with YFP-Cam cassette	8
tbl3a	High tolerance strain, evolved *in vitro* from KLY under transient antibiotic exposures of 3 hours (*vapB(V5E*) mutant)	8
tbl5a	High tolerance strain, evolved *in vitro* from KLY under transient antibiotic exposures of 5 hours (*metG(Y620N*) mutant)	8
MGY	MG1655 (seq) with YFP-Cam cassette	26
MGHY	*P1* transduction of high persistence *hipA7* mutation from HM22^14^ (tetracycline selection) into MGY	This work
MGZ1-PSA11	*E. coli* K-12 strain with pSA11 plasmid bearing ampicillin resistance cassette.	27
