# Supplementary material for: TDtest: easy detection of bacterial tolerance and persistence in clinical isolates by a modified disk-diffusion assay
Source: Sci Rep. 2017 Feb 1;7:41284. doi: 10.1038/srep41284 (PMC5286521; doi:10.1038/srep41284)
Supplement: Supplementary Figures and Movie [file srep41284-s1.pdf]

TDtest: easy detection of bacterial tolerance and persistence in clinical isolates by a modified disk-diffusion assay

Orit Gefen[1], Betty Chekol, Jacob Strahilevitz[3] and Nathalie Q. Balaban[1,2]

1 Racah Institute of Physics, Edmond J. Safra Campus, The Hebrew University of Jerusalem, Jerusalem 9190401, Israel.

2 The Harvey M. Kruger Family Center for Nanoscience and Nanotechnology, Edmond J. Safra Campus, The Hebrew University of Jerusalem, Jerusalem 9190401, Israel.

3 Department of Clinical Microbiology and Infectious Diseases, Hadassah-Hebrew University Medical Center, Jerusalem, 91120, Israel

Supplementary Figures and movie

Supplementary Fig. S1:

To test whether the late colonies appearing in the inhibition zone after the second step of the TDtest are growing simply because of the removal of the antibiotic disk, we compared the TDtest to various controls, using ampicillin (10 µg) and medium tolerance strain tbl3a: (A)TDtest. (B) Same as in (A) but without the addition of the glucose disk. (C) Same as (B) but without removing the antibiotic disk (see also Supplemental Fig 2). (D) TDtest. (E) Same as (D) but disk at step II contains only water (DDW), the glucose solution solvent.

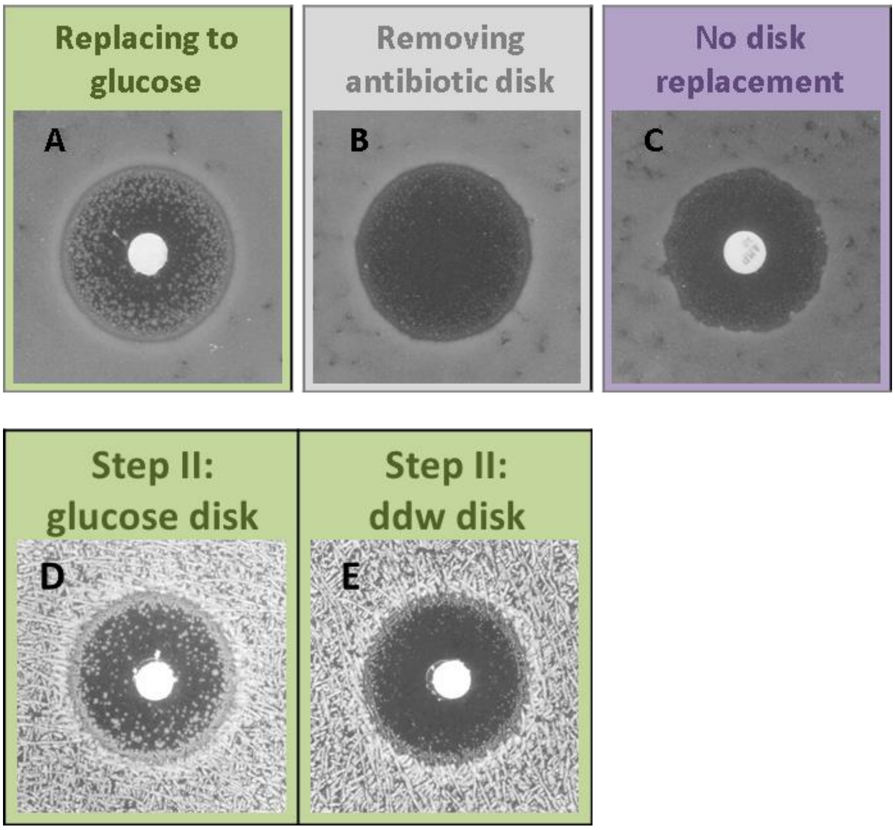

Supplementary Fig. S2:

Time-lapse imaging, using the Scanlag setup[1], of the TDtest versus the regular disk diffusion assay allows visualizing the effect of nutrient replenishment: (A) regular disk diffusion assay for ampicillin (10 µg) and strain B340 (*E.cloacae* isolate). (B) TDtest on same strain where the ampicillin disk is replaced with a glucose disk 18 hours after plating. Orange arrows mark the appearance of tolerant colonies in the TDtest.

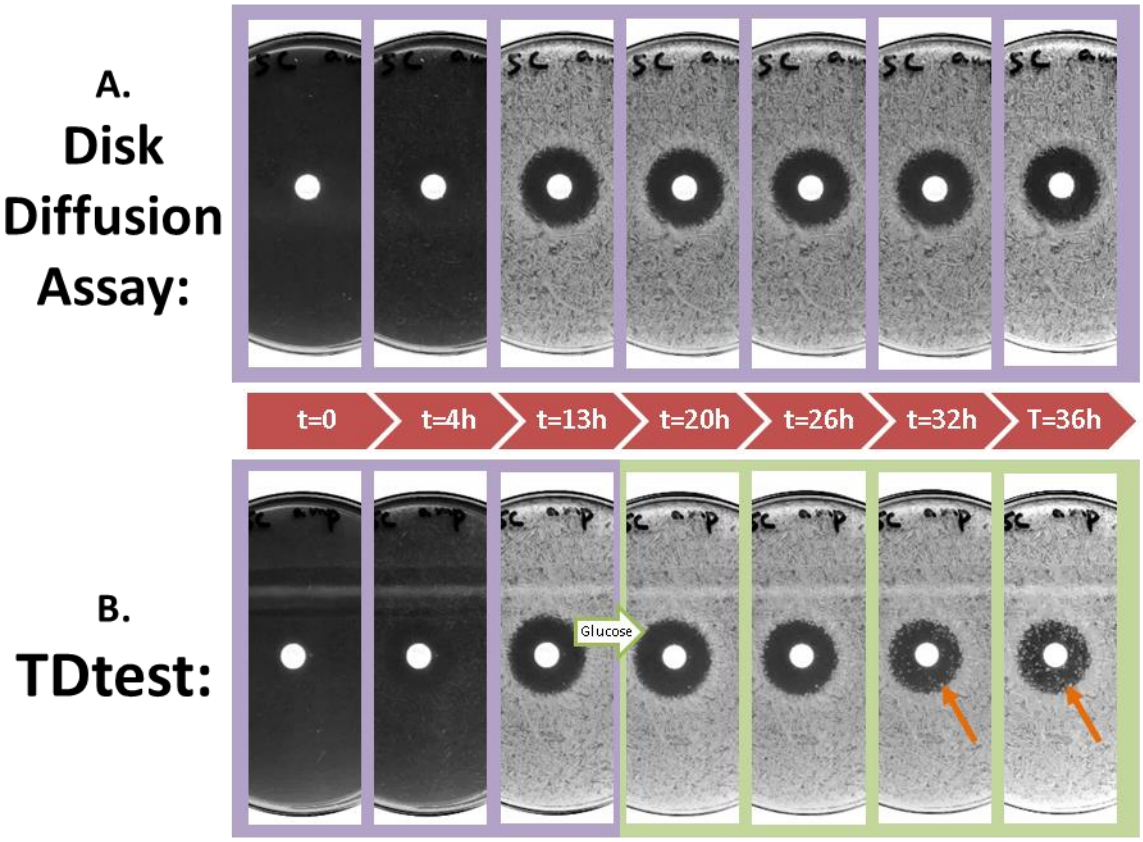

Supplementary Fig. S3:

Colonies detected with the TDtest are typically not due to resistant mutants. tbl3a was tested with imipenem (2.5µg) and ampicillin (10µg).

(A) The growth inhibition zone after the first step of the TDtest (exposure to antibiotics only). The dashed lines mark the diameter of the inhibition zone. (B) Growth of colonies inside the inhibition zone after the second step of the TDtest (replacement of the antibiotic disk with a glucose-containing disk). Appearance of colonies inside the inhibition zone occurs after a few hours and indicates tolerant/persistent bacteria. (C) A mixture of 5-10 colonies that grew inside the inhibition zone (panel B) were picked and retested to the same antibiotic as in panel A. The dashed lines mark the diameter of the inhibition zone, same as in panel A. (D1-D5) resuspension and retest of 5 different colonies inside the inhibition zone, marked as 1-5, respectively in B, shows again the same diameter as in A.

Note that sub cultures of the same strain may lose the tolerant phenotype, probably due to the strong dependence of tolerance on growth conditions.

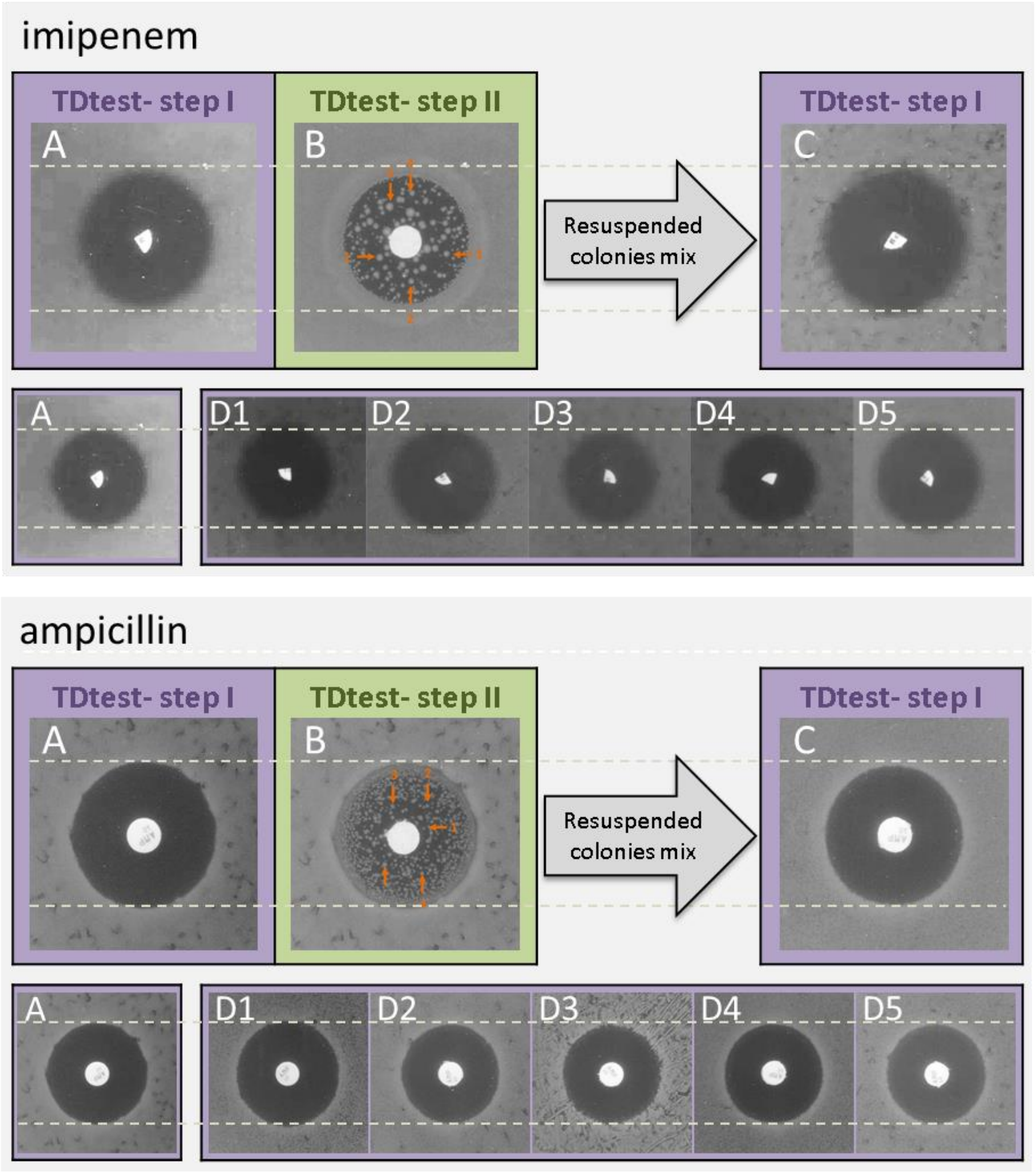

Supplementary Fig. S4:

A single colony was observed growing inside the inhibition zone in the first step of the TDtest with ampicillin. Very rarely for beta lactams (twice out of hundreds of assays), we were able to detect a single colony growing well inside the inhibition zone before the addition of glucose (marked with orange arrow), and was found to be resistant, i.e. MIC>50µg/ml. However, the large majority of colonies growing inside the inhibition zone after the second step of TDtest are not resistant (see Supplementary Fig. S3).

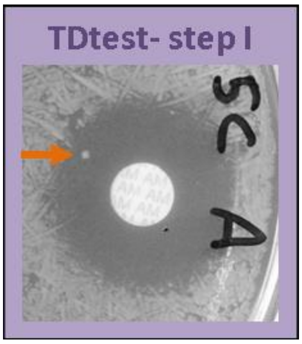

Supplementary Fig. S5:

Reproducibility of the TDtest.

(A-F) TDtest on tbl3a with 10 µg ampicillin for 3 biological replicates: (A-D) replica from the same experiment. (E-F) biological replicates from two additional independent experiments.

(G-H) TDtest on W547 with 0.25 µg ertapenem for two independent experiments.

(I-J) TDtest on U453 with 0.25 µg ertapenem for two independent experiments.

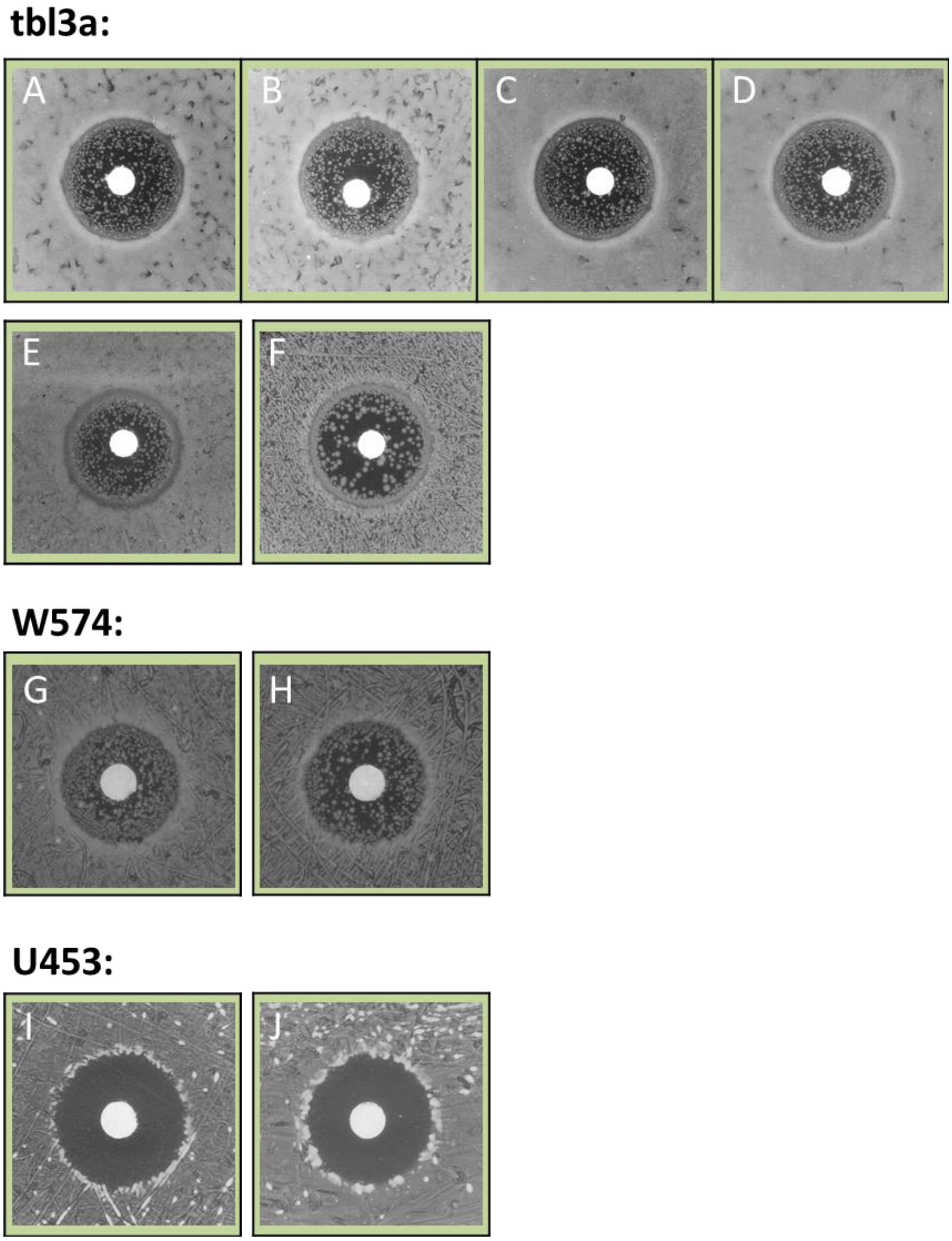

Supplementary Fig. S6:

The second step of TDtest of tbl3a, applied with several antibiotics from different classes:

Fluoroquinolone (ciprofloxacin), aminoglycoside (kanamycin), cephalosporin(cefazolin), carbapenems (ertapenem, imipenem) and penicillin (ampicillin).

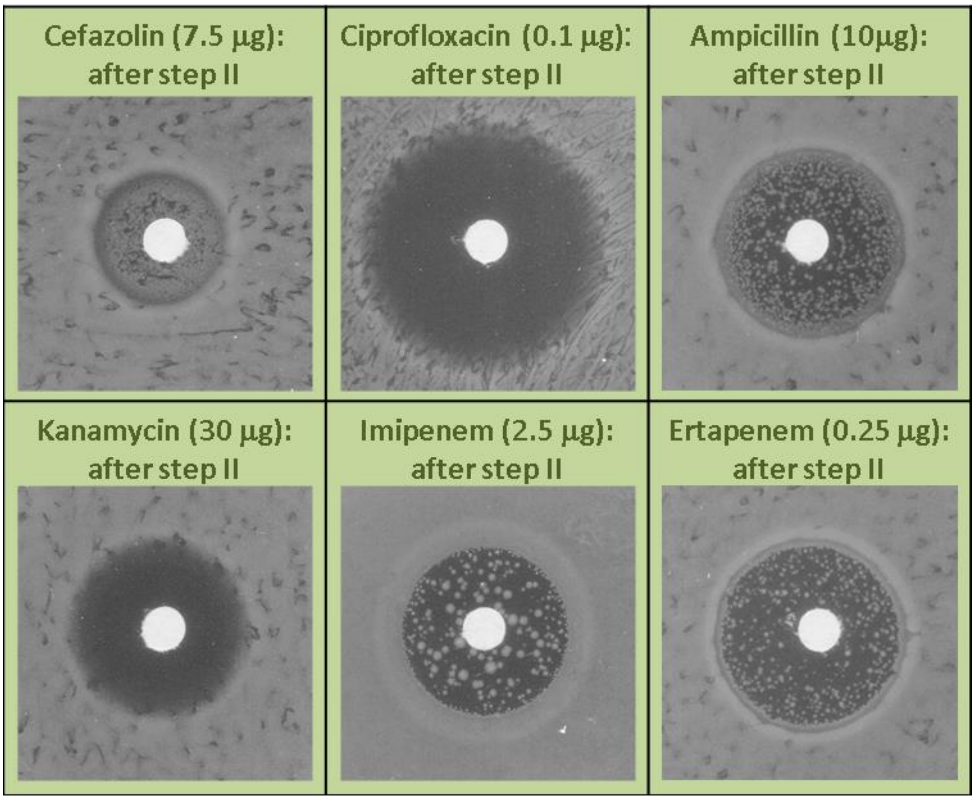

Supplementary Fig. S7:

TDtest for different amounts of antibiotics. The TDtest requires adjustment of the amount of antibiotic in the disk used in the first step, to ensure that it drops below the MIC of the bacteria tested after sufficient time. If the MIC is not known, different amounts should be tested (see Methods). In this example, TDtests to three different amounts of ertapenem were sufficient to reveal the medium tolerance of the tbl3a strain.

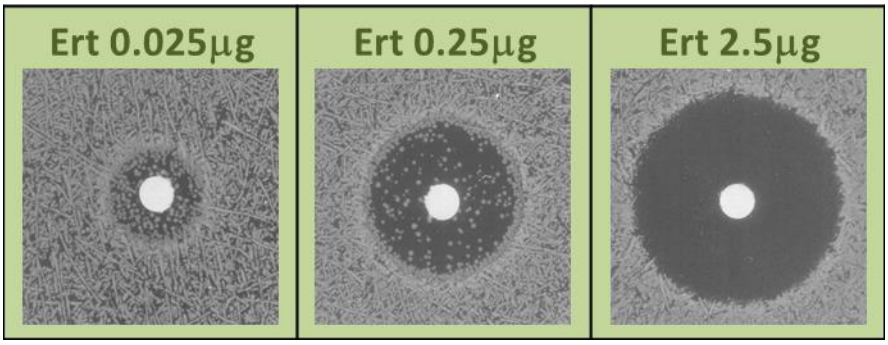

Supplementary Fig. S8:

TDtest of tbl5a for ampicillin (10 µg) on different microbiological media.

(A) LB agar plate. (B) Mueller-Hinton agar plate. (C) Mueller-Hinton+5% sheep blood agar plate.

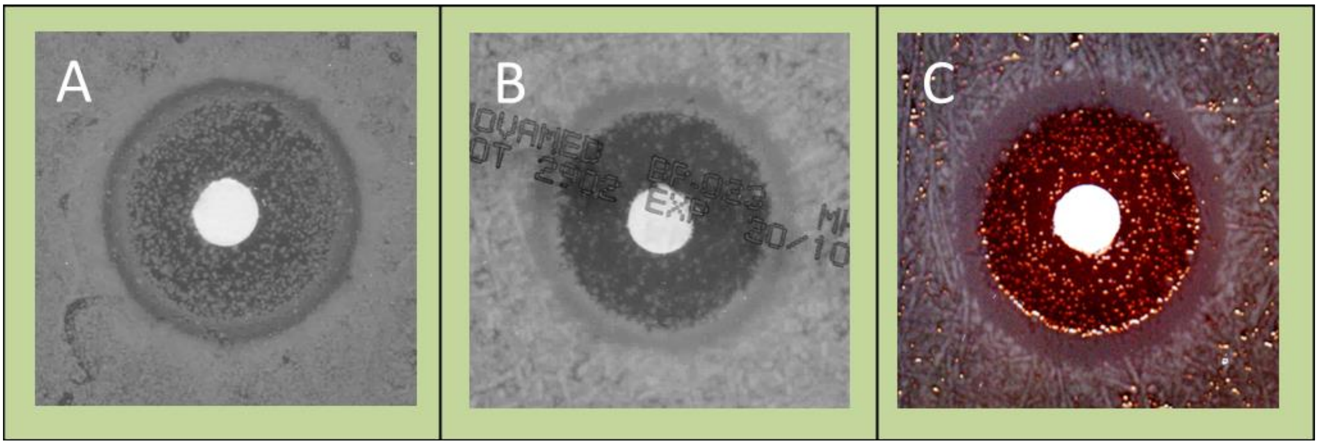

Supplementary Fig. S9:

Schematic plot of the antibiotic profile in the plate for 4 time points relevant for the TDtest, for a point source of antibiotic located at the center of the plate (Fig. 2 Eq. 1 with D= $8.5 \cdot 10^{-6} cm^2/sec$, d=0.4cm): At *t*=9 hours, R1, the radius of the inhibition zone is formed[2,3]. After *t*=18 hours, the nutrients added with the second disk start to spread in the inhibition zone, and surviving bacteria can grow between the edge of the inhibition zone (R1) and R2. After several more hours, the concentration of the antibiotic drops below the MIC all over the plate, and surviving bacteria can grow in whole inhibition zone.

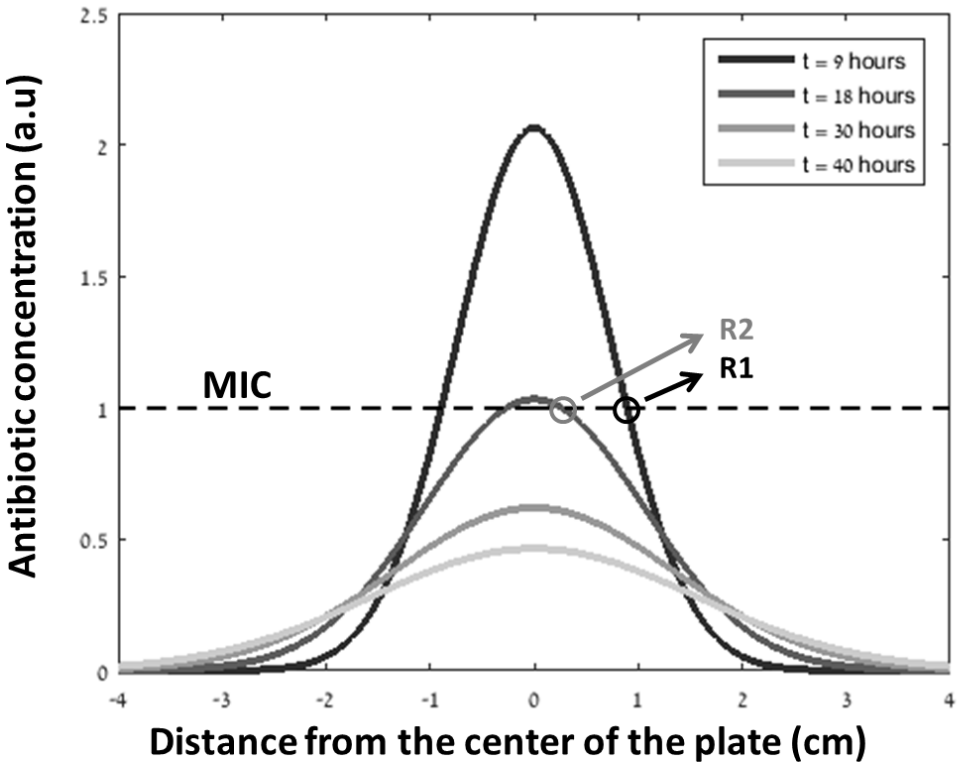

Supplementary Fig. S10:

We determined empirically that, as expected from the diffusion equation (Fig. 2 Eq. 1 & Fig. S9) during the second step of the TDtest, sensitive bacteria are able to grow within the inhibition zone formed around 7.5µg kanamycin disk, by streaking new bacteria inside the inhibition zone after 22 hours, simultaneously with the addition of glucose solution directly on the antibiotic disk. The growth of the streaks indicates that the antibiotic concentration falls below the MIC after this time. (bar: 1cm)

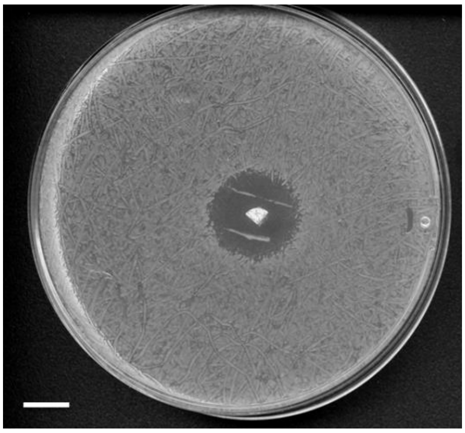

Supplementary Fig. S11:

Measured fluorescence levels of fluorescein over time for three locations in an agar plate. red line- data, blue line- fit using Eq.1. (A)r=1.7cm, (B)r=1.2 cm and (C) r=0.7 cm from the source (see Methods)

The diffusion coefficient for fluorescein at 37°C from the fit to the data is $D = 8.5 \cdot 10^{-6} \pm 9 \cdot 10^{-7}\ cm^2/sec$.

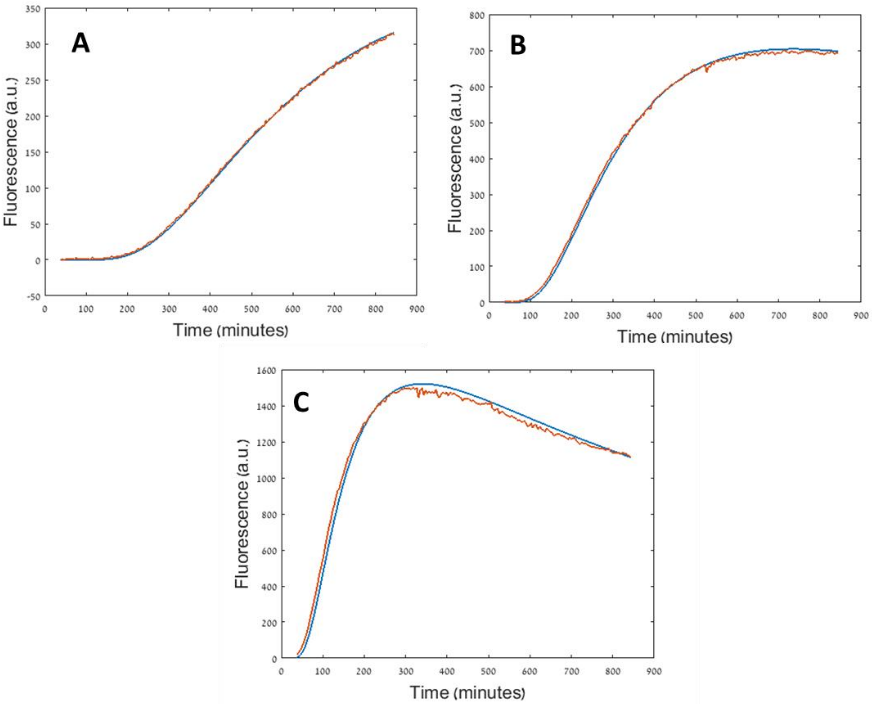

Supplementary movie S1

TDtest versus disk diffusion test

tbl3a bacteria growing on agar plates with ampicillin disk (10μg). The growth inhibition zone is similar in all three plates. The ampicillin disk in the left plate remains until the end of the experiment, the ampicillin disk in the middle plate is replaced by a glucose disk after 18 hours of incubation and the right disk contains late-release glucose from the beginning. Due to the glucose supply, surviving bacteria form colonies and can be seen inside the growth inhibition zone in both right and middle plates, while no visible colony can be seen inside the growth inhibition zone in the left plate, where glucose was not added. Movie duration: 35h.

Supplementary References

1 Levin-Reisman, I. *et al.* Automated imaging with ScanLag reveals previously undetectable bacterial growth phenotypes. *Nat Methods* **7**, 737-739, doi:10.1038/nmeth.1485 (2010).

2 Bonev, B., Hooper, J. & Parisot, J. Principles of assessing bacterial susceptibility to antibiotics using the agar diffusion method. *The Journal of antimicrobial chemotherapy* **61**, 1295-1301, doi:10.1093/jac/dkn090 (2008).

3 Ericsson, H., Tunevall, G. & Wickman, K. The paper disc method for determination of bacterial sensitivity to antibiotics. Relationship between the diameter of the zone of inhibition and the minimum inhibitory concentration. *Scandinavian journal of clinical and laboratory investigation* **12**, 414-422, doi:10.3109/00365516009065406 (1960).
